# A new species of *Neocarus* Chamberlin & Mulaik, 1942 (Opilioacarida, Opilioacaridae) from Brazil, with remarks on its postlarval development

**DOI:** 10.3897/zookeys.358.6384

**Published:** 2013-12-04

**Authors:** Leopoldo Ferreira de Oliveira Bernardi, Hans Klompen, Mauricio Sergio Zacarias, Rodrigo Lopes Ferreira

**Affiliations:** 1 Departamento de Biologia/UFLA, Lavras, Minas Gerais, Brasil; 2 Museum of Biological Diversity, Ohio State University, 1315 Kinnear Road, Columbus, Ohio 43212-1192, USA; 3 Embrapa Café, EPAMIG/EcoCentro, Lavras, Minas Gerais, Brasil; 4 Laboratório de Ecologia Subterrânea, Setor de Zoologia/Departamento de Biologia, UFLA, Lavras, Minas Gerais, Brasil

**Keywords:** Acari, Parasitiformes, growth, sexual dimorphism, Neotropical

## Abstract

*Neocarus proteus* **sp. n.**, is described from caves and the surrounding epigean environment of ferruginous outcrops (Cangas) in Minas Gerais, Brazil. In addition, some notes about development in this species are presented. *Neocarus proteus* is the only species in the genus that has smooth or barbed genital setae and that carries coronidia on the basitarsi, tibiae and genua of legs II–III. Females carry additional setae with rounded tips on the subcapitulum, and are, on average, larger than males. This distinct sexual dimorphism appears in the tritonymphal instar and is maintained in the adults.

## Introduction

Opilioacarida is one of the orders of mites (Lindquist et al. 2009), but has a very small number of described species. It currently contains one family (Opilioacaridae) with a total of 11 genera (*Adenacarus* Hammen, 1969, *Caribeacarus* Vázquez & Klompen, 2009, *Indiacarus* Das & Bastawade, 2007, *Neocarus* Chamberlin & Mulaik, 1942, *Opilioacarus* With, 1904, *Paracarus* Redikorzev, 1937, *Panchaetes* André, 1947, *Phalangiacarus* Naudo, 1963, *Salfacarus* Hammen, 1977, *Siamacarus* Leclerc, 1989 and *Vanderhammenacarus* Leclerc, 1989), comprising 34 extant and 2 fossil species. Even with this small number of described species, the geographical distribution of these mites is wide, extending to all the continents, except Antarctica. They have been reported from a total of 25 countries (United States, Mexico, Cuba, Puerto Rico, Nicaragua, Costa Rica, Panama, Venezuela, Brazil, Argentina, Uruguay, Italy, Greece, Algeria, Angola, Gabon, Madagascar, Ivory Coast, Tanzania, South Africa, Yemen, Kazakhstan, India, Thailand and Australia (Walter and Proctor 1998, Vázquez and Klompen 2002, Walter and Harvey 2009, Vázquez and Klompen 2009, Vázquez and Klompen 2010, Bernardi et al. 2012).

Nine of the 11 known genera of Opilioacaridae are present in the Old World, but it is the New World that has the largest number of described species, with a total of 15, belonging to the genera *Neocarus* (11 spp.) and *Caribeacarus* (4 spp.). In Brazil, research on these mites is still in its initial phase, and only three species are currently known, two belonging to the genus *Neocarus* (Hammen 1969, Bernardi et al. 2012), and one to *Caribeacarus* (Bernardi et al. 2013).

Even though Opilioacarida are considered a primitive group and hence of importance for understanding phylogenetic relationships among mites, current knowledge of Opilioacarida consists mainly of taxonomic descriptions, while studies on behavior, development and other aspects of their biology are rare (Grandjean 1936, Coineau and Legendre 1975, Hoffmann and Vázquez 1986, Vázquez and Palacios-Vargas 1988, Walter and Proctor 1998, Walter and Harvey 2009, Klompen 2000). The objective of this study is to add to the knowledge of both their taxonomy and development by describing a new species of Opilioacaridae found in Brazil, and providing notes on postembryonic development.

## Materials and methods

### Study area

**Figures 1–2.**
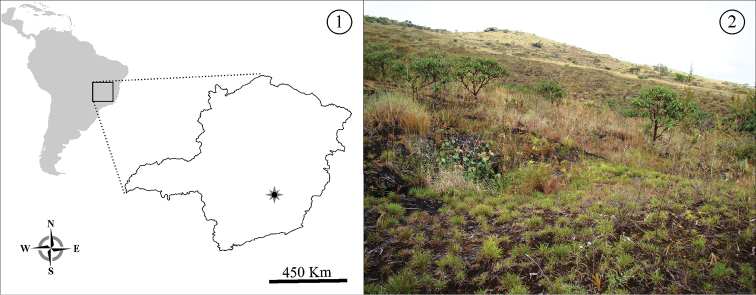
Collection site of *Neocarus proteus* sp. n. **1** Location in Minas Gerais, Brazil (star) **2** Detail of the canga environment.

All of the specimens used in the present work come from collections conducted in 2011 in caves and the epigean environment of ferruginous outcrops locally known as “Cangas”, in the Municipal district of Mariana, Minas Gerais, Brazil (Figs 1 and 2).

The Cangas represent an ancient terrain, with restricted distribution, and are composed of important iron ore deposits. In spite of being located inside the Atlantic Forest, the vegetation is different from that typically found in this biome, consisting mostly of open grasslands, herbaceous plants and sparsely distributed trees (Jacobi et al. 2007, Jacobi and Carmo 2008).

Floristic and subterranean biodiversity surveys conducted in this area suggest high species richness and a great number of endemic species. Unfortunately, the Cangas are increasingly targeted for intense mineral exploration that puts this biodiversity at risk, because the entire environment is modified during the ore extraction process (Ferreira 2005, Jacobi et al. 2007, Jacobi and Carmo 2008).

### Methods

Two different methods were used to collect specimens. The first involved active collection of specimens in the field, searching under stones, in organic matter accumulations and cracks in the soil. The second method was extraction from litter and soil using Berlese-Tullgren funnels (run time 72 hours, heat from a 25 watt bulb). All the specimens collected were stored in 80% ethanol in vials.

Most material was studied as slide-mounted specimens. For this purpose, specimens were dissected, cleared in Nesbitt’s solution and mounted on slides using Hoyer’s medium (Walter and Krantz 2009).

Specimens were studied with the aid of a Leica DMLS microscope and a Zeiss Axioscope 3 phase contrast microscope, equipped with a drawing tube. Photographs were made with the aid of Nikon Eclipse 90i automated DIC microscope with an integrated digital camera. Measurements were taken using an ocular micrometer and are presented as ranges in micrometers (μm). The nomenclature of setae and other morphological characters follows Grandjean (1936), Hammen (1969, 1977) and Vázquez and Klompen (2002, 2009). The terminology used for the sternal setae (*St1*, *St2*, *St3* and *St5*) follows an attempt to unify setal nomenclature for all Parasitiformes (H. Klompen and M. M. Vázquez, in prep.).

To recognized the instars, we used the criteria for Opilioacaridae provided by Coineau and Hammen (1974), as confirmed by Klompen (unpublished data). These characteristics are: protonymph with 2 pairs of stigma, no acrotarsus on legs II–IV, only 3 pairs of setae on sternal area; deutonymphs with 3 pairs of stigma, acrotarsus present on legs II–IV, usually with more than 3 pairs of setae on sternal region; tritonymphs with 4 pairs of stigma, a divided trochanter, but no sexual organs (ovopositor and male sexual gland absent) are present; in adults sexual organs are present.

Instar abbreviations used: PN = protonymph; DN = deutonymph; TN = tritonymph; F = female; M = male.

Collection sites of the specimens examined were georeferenced using coordinates in degrees, minutes and seconds with the South American Datum (SAD 69) geodesic system.

Specimens are deposited at the Mite Reference Collection, Department of Entomology and Acarology, Escola Superior de Agricultura “Luiz de Queiroz” (MZLQ), Universidade de São Paulo, Piracicaba–SP, Brazil; Collection of Subterrean Invertebrates (ISLA), Section of Zoology de Zoologia, Department of Biology, Universidade Federal de Lavras, Lavras–MG, Brazil; and Ohio State University Acarology Collection (OSAL), Museum of Biological Diversity, Columbus, OH, USA.

### Statistical analysis

To determine the presence of the sexual dimorphism in size for tritonymphs and adults we conducted a series of generalized linear models (GLMs) based on total length of legs I–IV. These analyses were conducted using R PROGRAM (R Development Core Team 2010).

## Results

### Taxonomy
Family Opilioacaridae With, 1904
Genus *Neocarus* Chamberlin & Mulaik, 1942

#### Neocarus proteus sp. n.

http://zoobank.org/A94A5CD4-8833-4692-807C-572DD71ECF28

http://species-id.net/wiki/Neocarus_proteus

##### Type material.

**Holotype female. Brazil: Minas Gerais: Mariana municipality,** 20°20'49.3"S, 43°26'50.8"W, epigean, coll. Pellegrini TG, Souza MFVR, Silva MS, Pompeu DC and Ferreira RL, 21.XII.2011 (**MZLQ**).

*Paratypes*: Brazil: Minas Gerais: Mariana municipality, 20°20'57.1"S, 43°26'37.5"W, coll. Pellegrini TG, Souza MFVR, Silva MS, Pompeu DC and Ferreira RL, 17.XI.2011, 2M (**ISLA**); same locality and collectors: 20°20'57.1"S, 43°26'37.5"W, 14.XI.2011, 1TN, 2F, 6M (**OSAL**); 20°20'53.6"S, 43°26'47.6"W, 18.XI.2011, 1DN, 1TN, 2F, 1M (**MZLQ**); 20°20'57.3"S, 43°26'32.7"W, 19.XI.2011, 1PN, 2DN, 1F, 2M (**OSAL**); 20°20'49.3"S, 43°26'50.8"W, 21.XII.2011, 1F (**ISLA**); 20°20'57.5"S, 43°26'37.2"W, 25.XI.2011, 2PN, 6DN, 10TN, 5F, 6M (**ISLA**); 20°20'57.1"S, 43°26'37.5"W, 14.XII.2011, 2F, 7M, 1TN (**ISLA**) (Table 1).

**Table 1.** Records of *Neocarus proteus* sp. n. in Mariana, Minas Gerais State, Brazil.

Stages	Mariana – Minas Gerais locality		
	20°20'57.1"S, 43°26'37.5"W	20°20'49.3"S, 43°26'50.8"W	20°20'53.6"S, 43°26'47.6"W
PN	3	–	–
DN	8	–	1
TN	12	–	1
♀	10	2	2
♂	24	–	1

##### Differential diagnosis.

The presence of setae on the female pregenital area may be unique for the South American species of *Neocarus*, *Neocarus platensis* (Silvestri, 1905), *Neocarus potiguar* Bernardi et al., 2012, and *Neocarus proteus*. The female genital area is nude in all *Neocarus* described from the USA, Mexico and Cuba. Unfortunately, the description of *Neocarus ojastii* Lehtinen, 1980 did not include details on the sternal or genital regions.

*Neocarus proteus* is the first species in the genus that is known to have the genital setae in the female adult variable, between weakly barbed and smooth. Furthermore, the present species is exceptional in carrying coronidia on the basitarsus, tibia and genu of legs II–III, and on the basitarsus and tibia of leg IV. In *Neocarus potiguar* the genital setae are smooth and the coronidia are limited to basitarsi II–IV. In *Neocarus platensis*, as redescribed by Hammen (1969), the genital setae appear smooth, with coronidia limited to the basitarsus and tibia of legs III (data on legs II and IV were not presented). The presence of coronidia on the tibiae and genua is unusual for *Neocarus*: in most species coronidia are restricted to basitarsi II–IV. Notably, in differentiating the genus *Panchaetes* from *Salfacarus*, Hammen (1977) listed the presence of coronidia on tibiae II–IV as unique to *Panchaetes*. Because this character is no longer restricted to *Panchaetes*, its separation from *Salfacarus* (the other African genus with numerous opisthosomal setae), may need to be revaluated.

The presence of six foliate (*d-type*) setae on the palp tarsus of most adults of *Neocarus proteus* (83.5% of females, 50% of males; N = 20) is uncommon within *Neocarus*. Two males have 5 setae on one palp and 6 on other palp. Of the 13 species of *Neocarus* currently described, only four; *Neocarus proteus* sp. n., *Neocarus nicaraguensis* Vázquez & Klompen, 2002, *Neocarus platensis* and *Neocarus potiguar*, carry five or six *d*-type setae on the palp tarsus; the other nine species carry only four or five.

Identifications of *Neocarus* species generally requires consideration of multiple characters simultaneously. Table 2 summarizes this type of comparative data.

**Table 2.** Comparative setal patterns for the pregenital and genital region of *Neocarus* adults.

Occurence	Species/Subspecies	Female		Male		Palp	
		Pregenital region	Genital region	Pregenital region	Genital region	*ch-* type	*d-* type
		No. and type of setae	No. and type of setae	No. and type of setae	No. and type of setae		
**North America**							
USA	*Neocarus texanus*	2 st/r	0	4–5 st/r	8–9 sh	14(21^a^)	5
Mexico	*Neocarus nohbecanus*	0	0	4-5 st/r	5-7st/r	17-19	4
Mexico	*Neocarus siankaanensis*	0	0	2 st/r	4 st/r	14-15	5
Mexico	*Neocarus bajacalifornicus bajacalifornicus*	2 st/r	0	5–8(13^a^) st/r	7–8(11^a^) st/r	14-18 (21^a^)	5
Mexico	*Neocarus bajacalifornicus chamelaensis*	2–3 st/r	0	4–5 st/r	4–6 st/r	16	5
Mexico	*Neocarus calakmulensis*	2–3 st/r	0	2–6 st/r	3–8 st/r	17	5
Mexico	*Neocarus veracruzensis*	2 st/r	0	6–8 st/r, 0–1 s	6–8 st/r	13	5
**Central America**							
Nicaragua	*Neocarus nicaraguensis*	2–5 st/r	0	2–7 st/r	3–6 st/r	18-22	5 or 6
Cuba	*Neocarus orghidani*	0	0	4–5 st/r	5–7 st/r	20-24	4
**South America**							
Venezuela	*Neocarus ojastii*	0	0	6–9 ?	13 st/r	-	-
Brazil	*Neocarus proteus*	2–5 st/r	4–6 wb	2–5 st/r	3–5 sh	12 or 13	5 or 6
Brazil	*Neocarus potiguar*	1 tp/r	4–8 sh	5 st/r	7–10 st/r	25-27	5 or 6
Brazil/Argentina/Uruguay	*Neocarus platensis*	0–2 st/r	6–9 sh	6–10 st/r	5–10 sh	14	5 or 6

^a^ - number of setae in super adults; sh: smooth setae; st/r - stout and ribbed setae; tp/r: tapering and ribbed setae; wb: weakly barbed setae.

##### Description.

**Figure 3.**
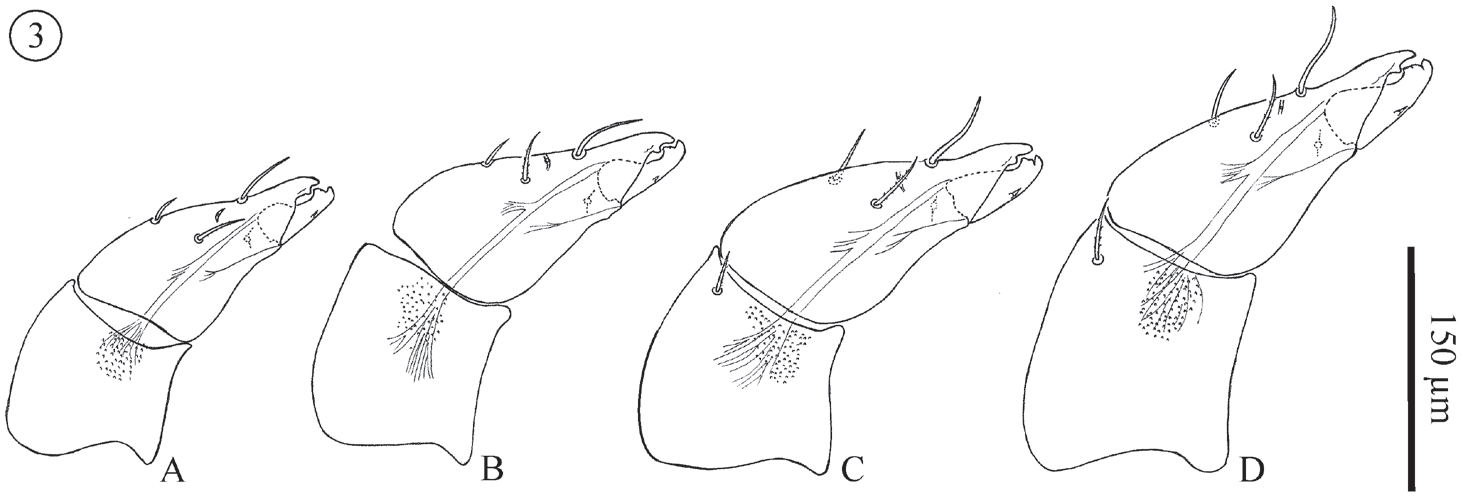
*Neocarus proteus* sp. n. Lateral view of chelicerae. **A** protonymph **B** deutonymph **C** tritonymph **D** adult female.

*Chelicera* (Fig. 3); Movable digit (PN 36.9–42.5; DN 45.8–50; TN 45.3–65.9; M 59–64.7; F 63–76.7), digit part of fixed digit (PN 33.6–40.1; DN 40.5–46.8; TN 45.3–61.3; M 45–52.2; F 52–67.5;) and entire fixed digit (segment) (PN 117–118.8; DN 141.5–156.3; TN 153.6–197.6; M 182–202.9; F 195.3–220). Basal segment without setae in all proto- and deutonymphs. One weakly barbed dorsal seta added in all adults and most tritonymphs (one specimen without setae and another 3 with a single seta on one of the two cheliceral bases). Fixed digits each with 2 smooth and 1 barbed seta in all instars (barbs on barbed setae more distinct in later instars). Dorsal and antiaxial lyrifissures present. Rounded and distinct teeth on the fixed digit, one large and distinct tooth with a small medial groove on movable digit. Both digits with a well developed terminal hook. Movable digit with one small denticle on its ventral margin in all instars, more distinct in later instars.

**Figures 4–5.**
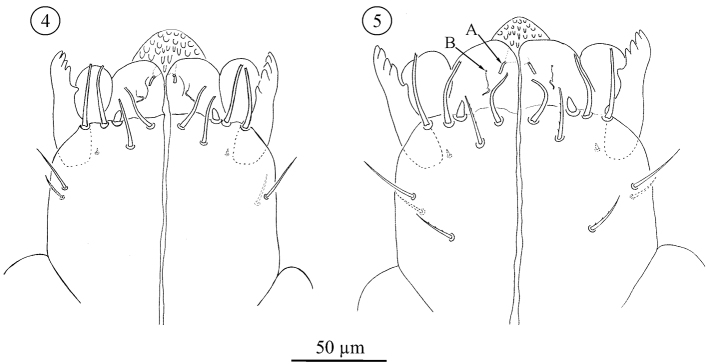
*Neocarus proteus* sp. n. Ventral view of subcapitulum. **4** protonymph **5** deutonymph. Arrows indicate the labial glands, **A**: *ogl1* and **B**: *ogl2.*

**Figures 6–7.**
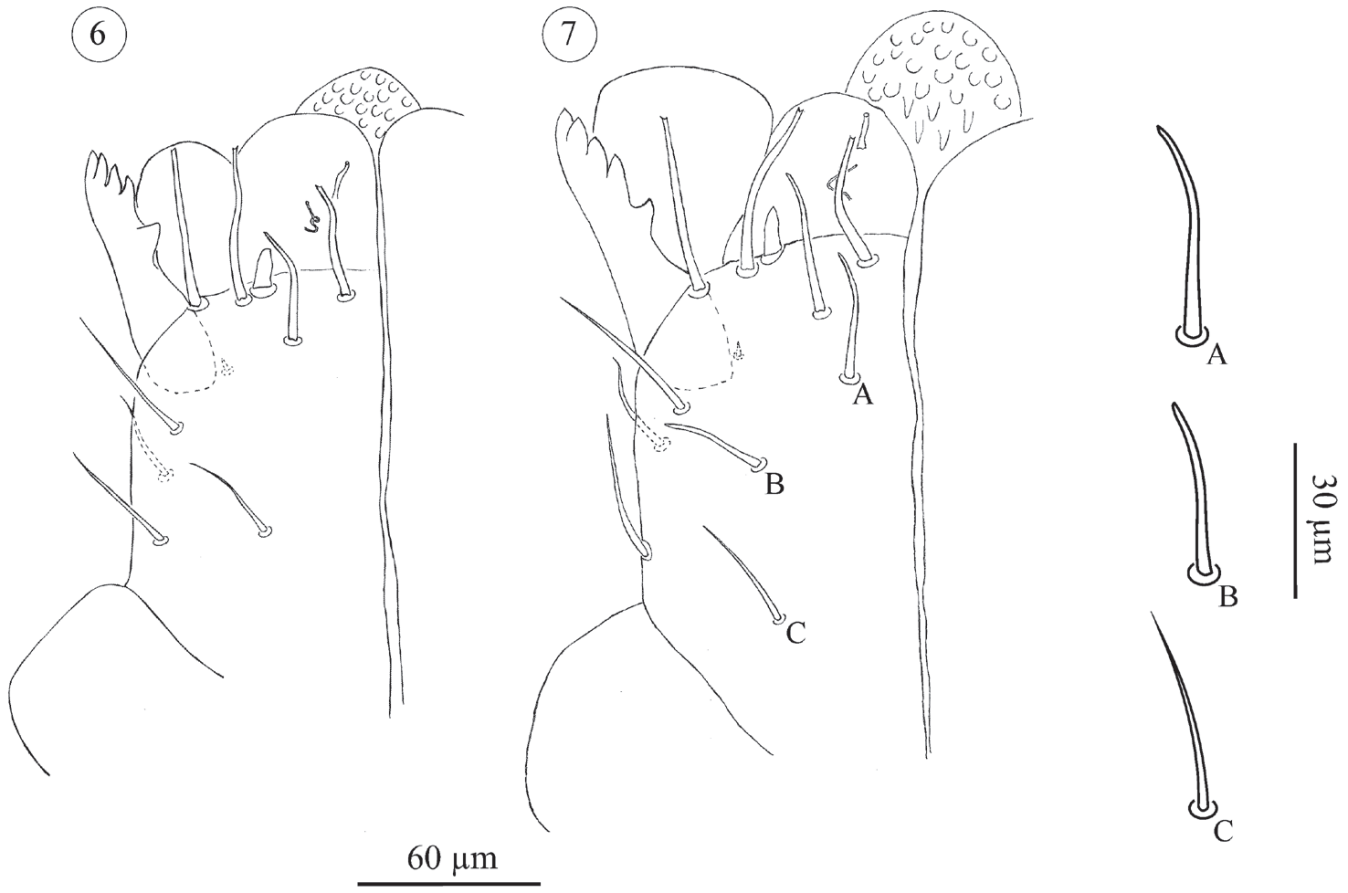
*Neocarus proteus* sp. n. Ventral view of subcapitulum. **6** male tritonymph **7** female tritotonymph. **A** and **B**: subcapitular setae with rounded tip; **C**: subcapitular setae with fine tip.

**Figures 8–9.**
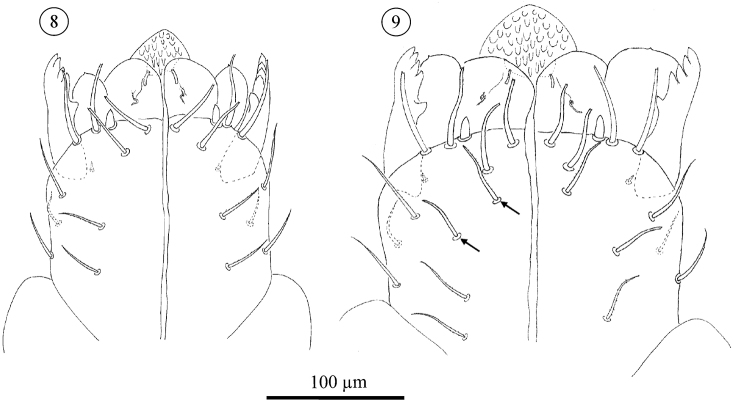
*Neocarus proteus* sp. n. Ventral view of subcapitulum. Arrows indicate the setae present in female tritonymphs and female adults only. **8** adult male **9** adult female.

*Subcapitulum* (Figs 4–9); All stages studied with 4 paralabial setae: *pl1* small, conical; With’s organ (*pl2*) membranous, discoid; rutellum (*pl3*) with 1 row of 5 teeth, inserted dorso-laterally; *pl4* small but distinct, inserted dorsal on subcapitulum. Lateral lips with two distinct canals, *ogl1* thicker and shorter than *ogl2* (Fig. 5). All instars also carry at least four circumbuccal setae, somewhat rod-like and with rounded tips. Female tritonymphs and females carry an additional two subcapitular setae resembling circumbuccal setae (Figs 7, 9), one smaller and smooth, the other bigger and weakly barbed, but both with a blunt, slightly rounded or bifurcate tip. These setae are either absent or more hair-like, smooth, tapering, and with a fine tip, in male tritonymphs and males. In those instars they resemble the remaining subcapitular setae.

The number of remaining subcapitular setae increases over ontogeny. All instars carry a single dorsal seta, smooth, tapering and with a fine tip. Ventrally, protonymphs, deutonymphs, male tritonymphs, and adult males carry, respectively, 1, 1–2, 2–4, and 4–6 similar fine-tipped setae (Figs 4–6, 8). Female tritonymphs and females carry 3–5 and 4–6 fine-tipped setae ventrally (Figs 7 and 9).

**Figures 10–12.**
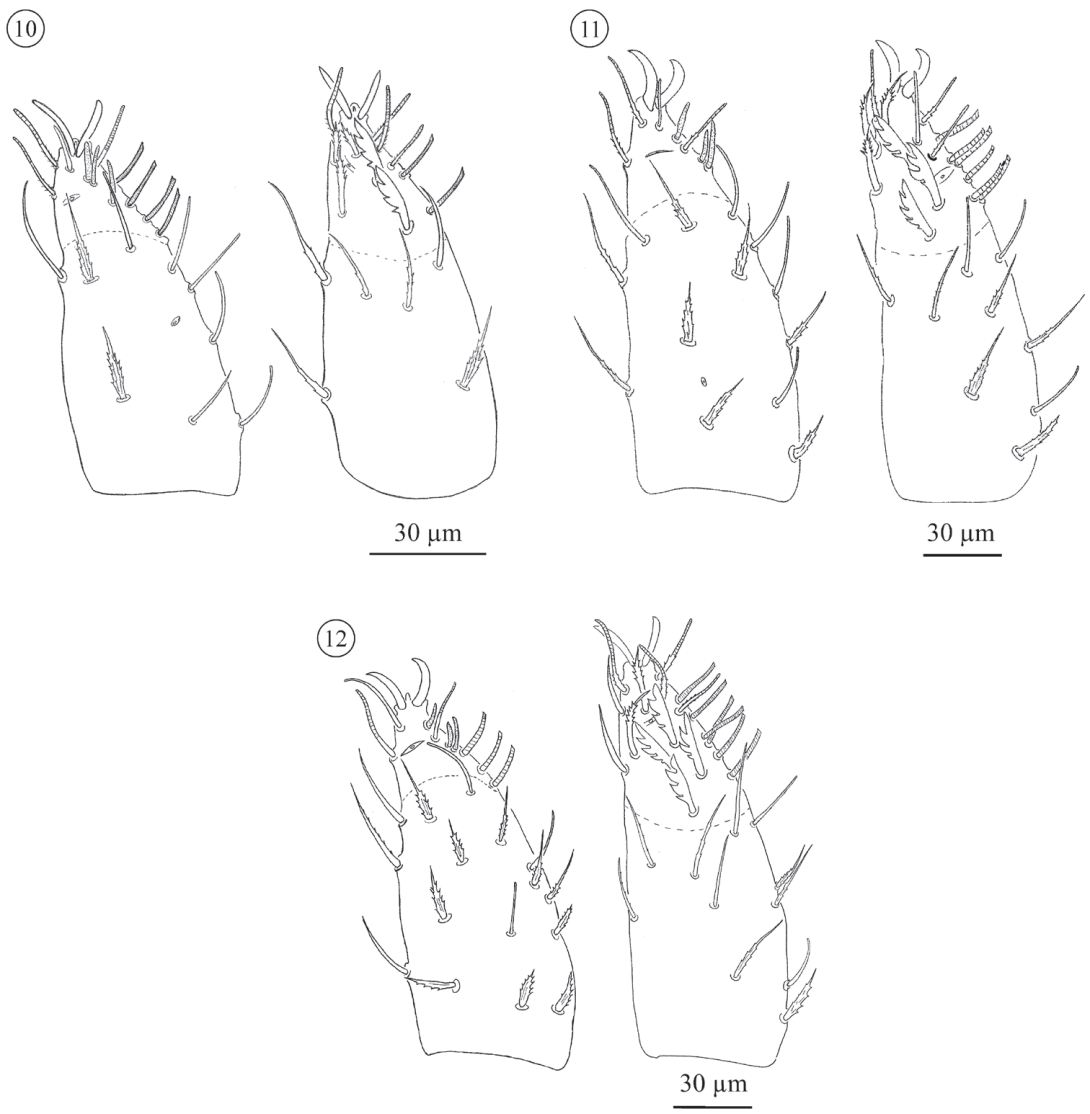
*Neocarus proteus* sp. n. Palp, ventral (left) and dorsal (right) view. **10** protonymph **11** deutonymph **12** tritonymph.

*Palp* (Figs 10 and 14). Adult trochanter with 3 to 4 ribbed, tapering (= r-type) setae; femur with 6–10 papilliform (= p-type) and 6–13 r-type setae; genu with 1–4 p-type and 27–45 r-type setae. Tibia and tarsus partially fused. Tibia with 6 smooth (= s-type) and 26–34 r-type setae. Palp tarsus with lyrifissures *i*π and *i*α. Setation includes 3 *s*, 5 or 6 *d*, 6 *v*, 17 *ch*, and 10–11 *sm* setae. The *sm3*-type seta is not present on the male palp. Pretarsus with a pair of well developed sessile claws. No distinct sexual differentiation observed; males generally with fewer trochanteral setae, but ranges overlap. Palp setation of immatures: trochanter: PN 0; DN 0–1; TN 2–3; femur: PN 5 r- and 1 p-type seta; DN 2–6 r plus 1–2 p; TN 4–9 r plus 3–5 p; genu: PN 6 r-type; DN 6–9 r- plus 0–1 p-type; TN 13–21 r- plus 1–2 p. Tarsi of proto-, deuto-, and tritonymphs with, respectively, 2, 3, and 4 *d* setae. Setation of tibiae not scored for immatures.

**Figures 13–14.**
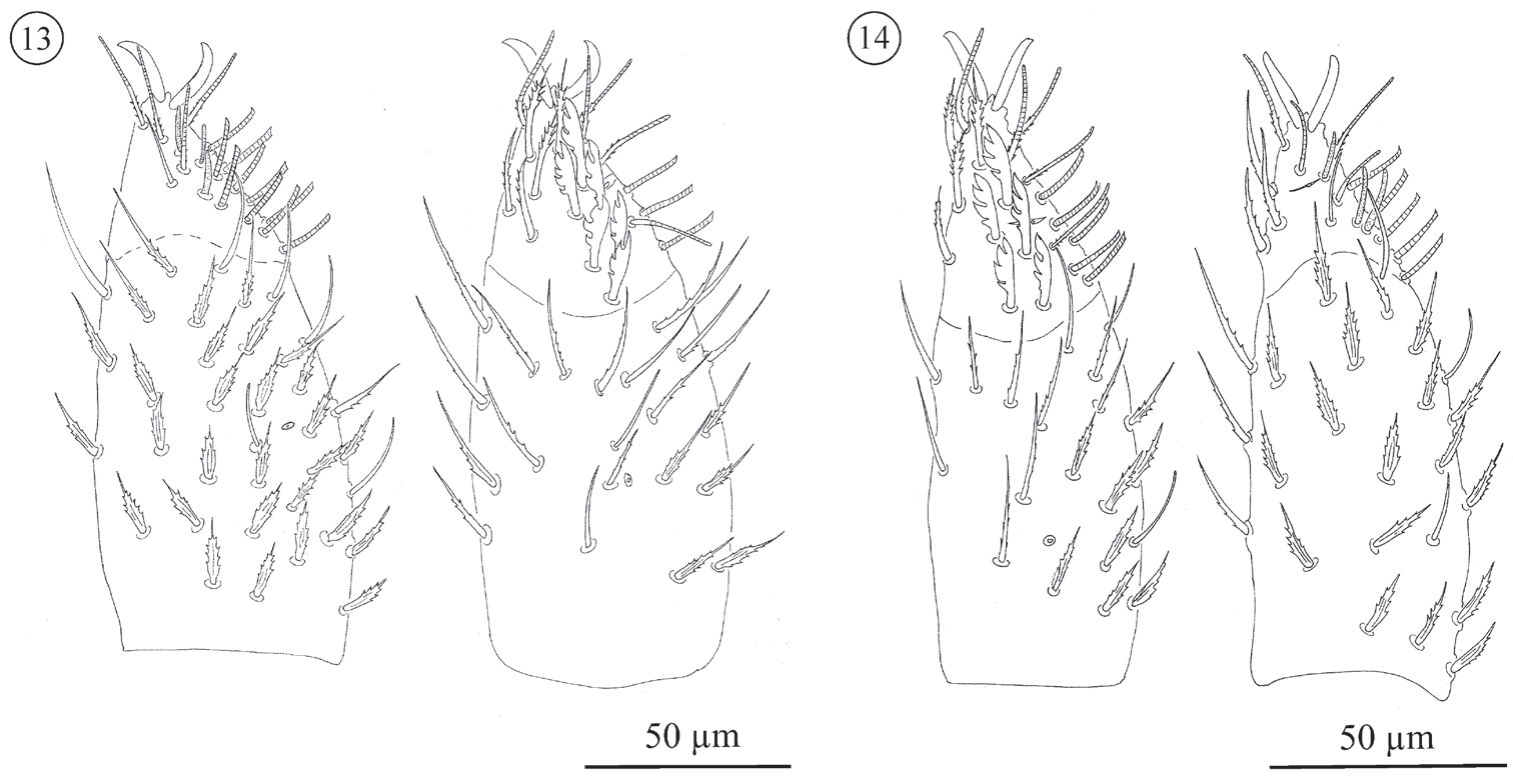
*Neocarus proteus* sp. n. Palp, ventral (left) and dorsal (right) view. **13** female **14** male.

**Figures 15–16.**
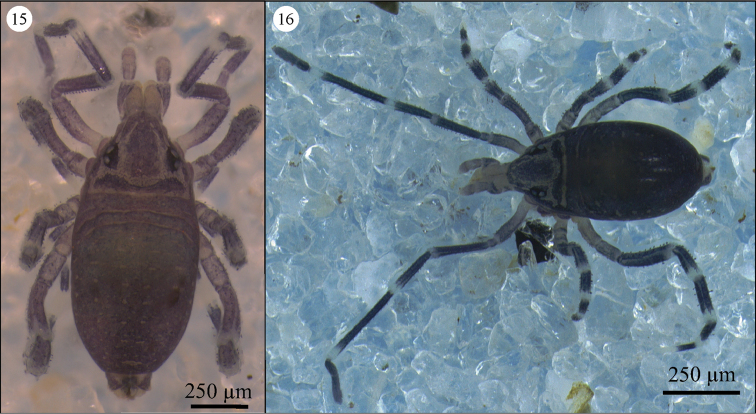
*Neocarus proteus* sp. n., adult female. Dorsal view.

*Idiosoma* (Figs 15 and 16). Adults with body longer (1230–1310 µm) than wide (450–630 µm), oval-shaped. Color light with dark blue patches. Body sometimes with a brownish background resulting from ingested food. Leg segments with strong violet banding.

*Dorsum*: Anterior dorsal shield in all stages with two pairs of eyes, and stout, ribbed setae. Dorsal idiosoma, between the shield and preanal segment, without setae, but with numerous lyrifissures arranged in transverse rows. Preanal segment with 1 dorsal and 2 ventrolateral stout, ribbed setae; anal plates in adults each with 10–15 stout, ribbed setae. Anal plates of PN, DN and TN each with, respectively, 1–2, 3–7 and 4–12 setae.

*Sternapophyses*: all stages studied with two setae, one small seta at the tip and one long, barbed seta positioned more basally.

**Figures 17–18.**
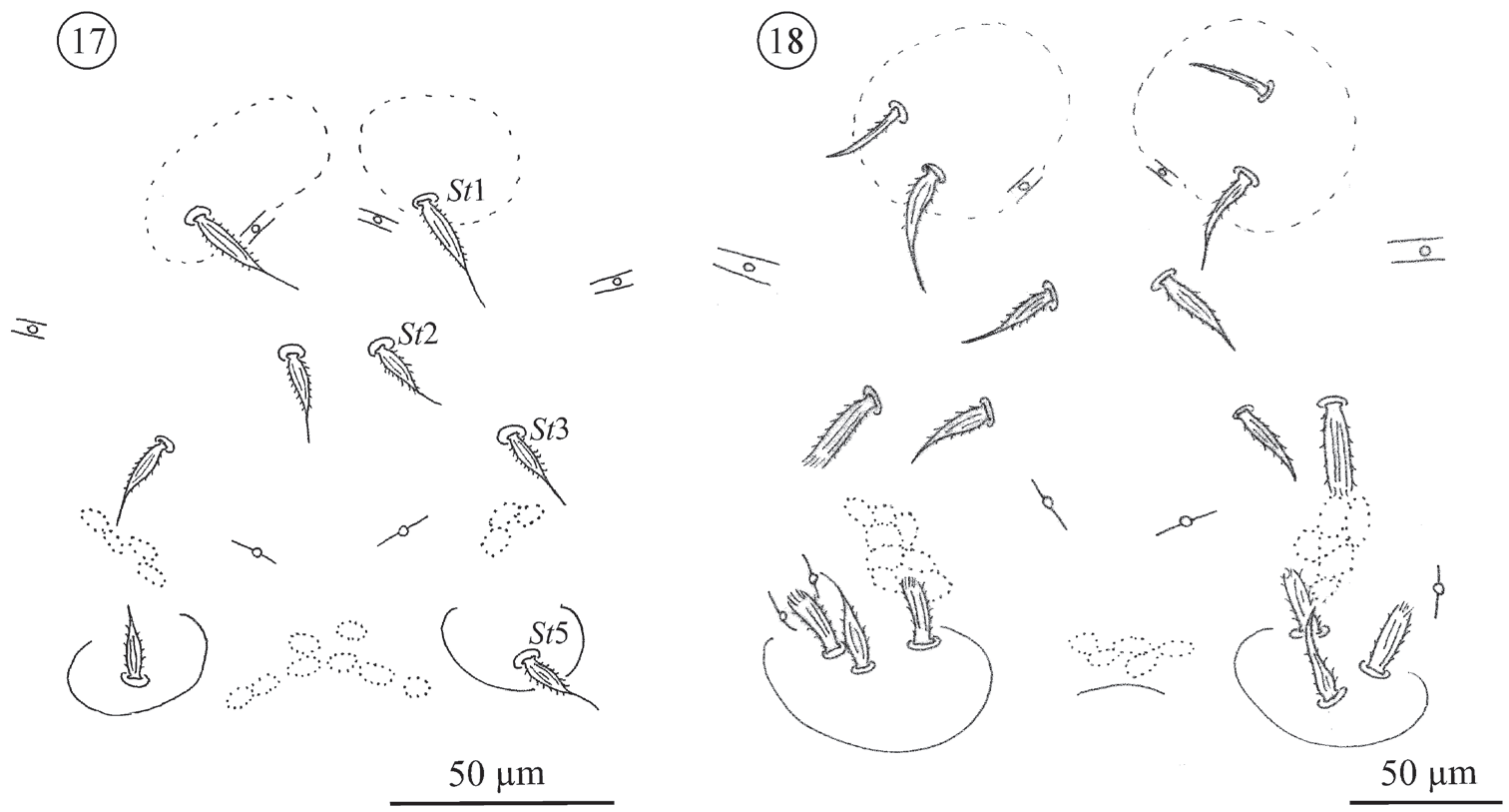
*Neocarus proteus* sp. n., sternogenital region. **17** protonymph **18** deutonymph.

*Sternogenital region in protonymphs* (Figs 17 and 26): sternal area with one pair of verrucae, each carrying one barbed, tapering seta (*St1*). Remaining sternal area with two pairs of small setae, both barbed and tapering (*St2*, *St3*). Pregenital area with one pair of pregenital capsule, each carrying one barbed, tapering seta (*St5*). Genital opening absent. With three pairs of lyrifissures, two pairs large, the third smaller, resembling the lyrifissures on the opisthosoma.

*Sternogenital region in deutonymphs* (Figs 18 and 26): sternal area with one pair of verrucae, each carrying one barbed, tapering seta (*St1*) and 0–1 barbed, fine setae. Remaining sternal area with two pairs of barbed, tapering setae (*St2*, *St3*), and 0–2 pairs of stout, ribbed and barbed setae usually positioned more laterally. Pregenital area with one pair of capsules, each carrying one barbed, tapering (*St5*) and 0–2 stout, ribbed and barbed setae. Genital opening present or absent, when present very small and poorly visible.

**Figures 19–20.**
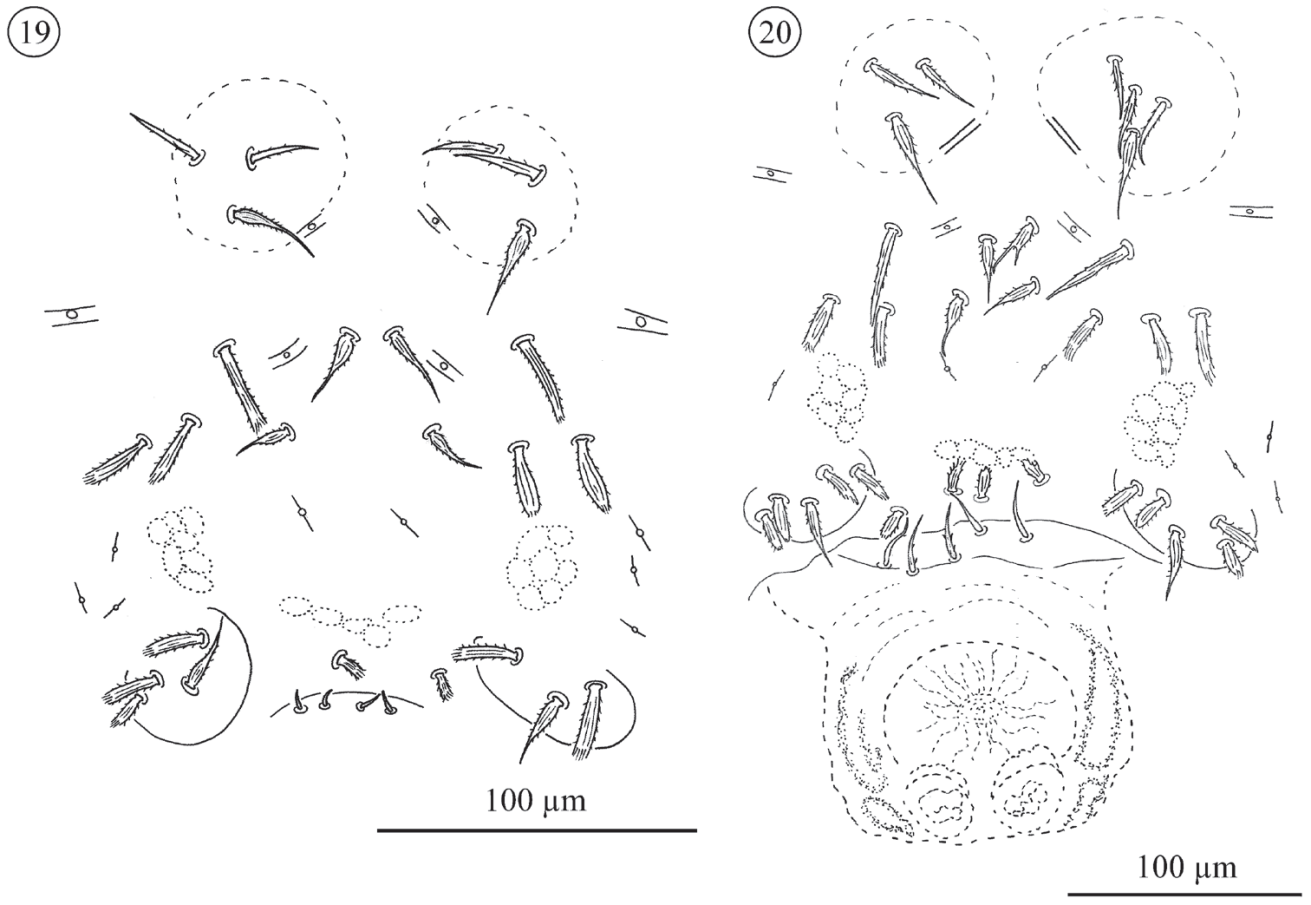
*Neocarus proteus* sp. n., sternogenital region **19** tritonymph **20** adult female.

*Sternogenital region in tritonymphs* (Figs 19 and 26). Sternal verrucae each with one barbed, tapering seta (*St1*) and 1–2 shorter, barbed, fine setae. Sternal area with two pairs of barbed, tapering (*St*2, *St*3) and 1–3 pairs of stout, ribbed and barbed seta. Each pregenital capsules with one barbed, tapering (*St*5) and 1–3 stout, ribbed and barbed setae. Pregenital area between capsules with 0 to 2 stout, ribbed and barbed setae. Genital opening present or absent. The genital area carries 0–4 small and fine setae.

**Figures 21–22.**
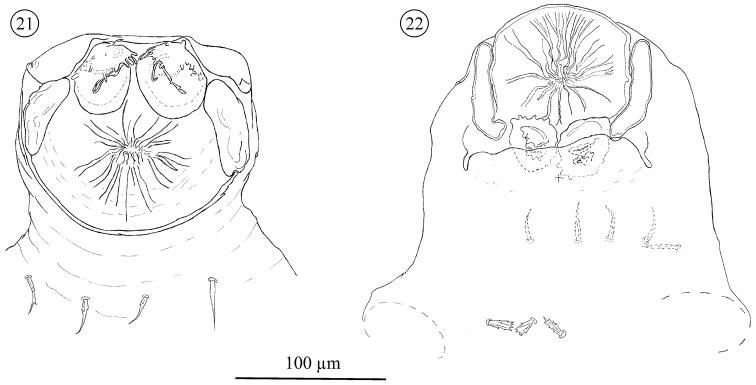
*Neocarus proteus* sp. n., Ovopositor. **21** invagined **22** evaginated.

**Figures 23–24.**
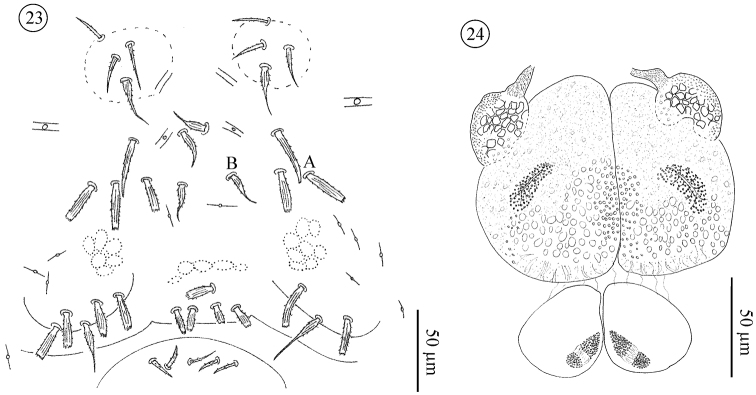
*Neocarus proteus* sp. n., adult male **23** sternogenital region **24** male genital glands. **A** and **B** indicate setae illustrated in Fig. 25.

**Figure 25.**
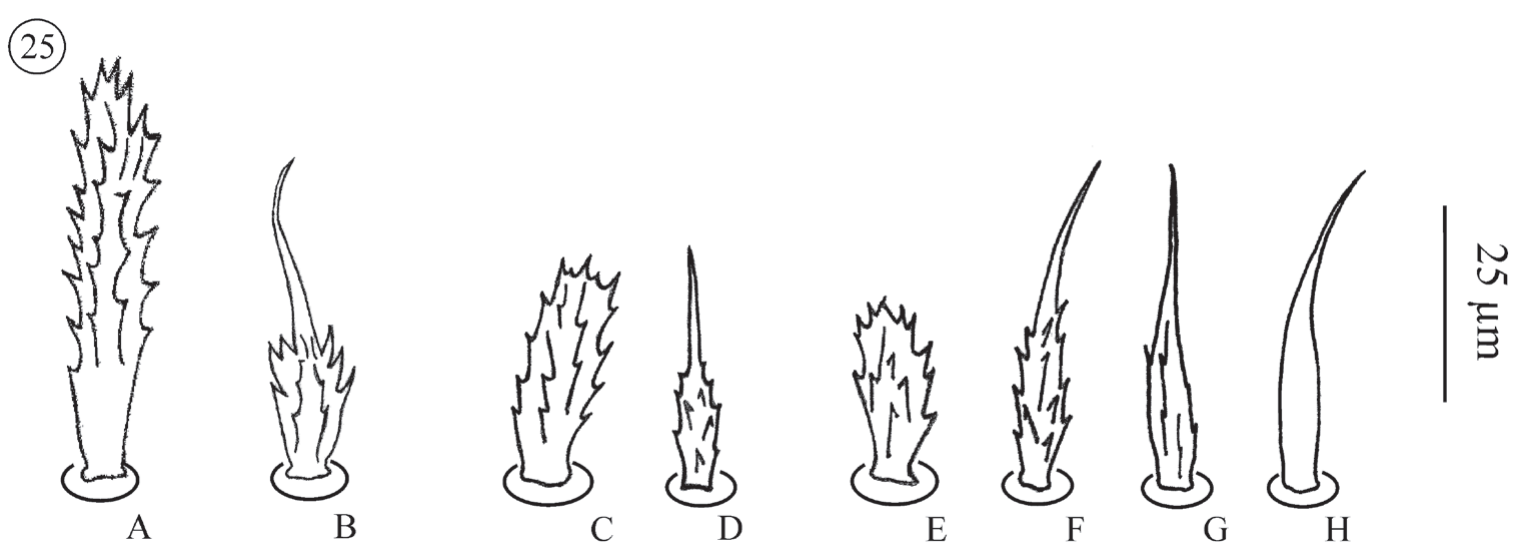
*Neocarus proteus* sp. n., details of shape of sternal and genital setae **A** stout and ribbed seta (sternogenital region) (see Fig. 23: **A**)**B** barbed and tapering seta (*St*3) (see Fig. 23: **B**) **C** stout and ribbed seta (male pre-genital region) **D** barbed and tapering seta (male genital region) **E** stout and ribbed seta (female pre-genital region) **F** and **G** barbed and tapering setae (female genital region) **H** smooth and tapering seta (female genital region).

**Figure 26.**
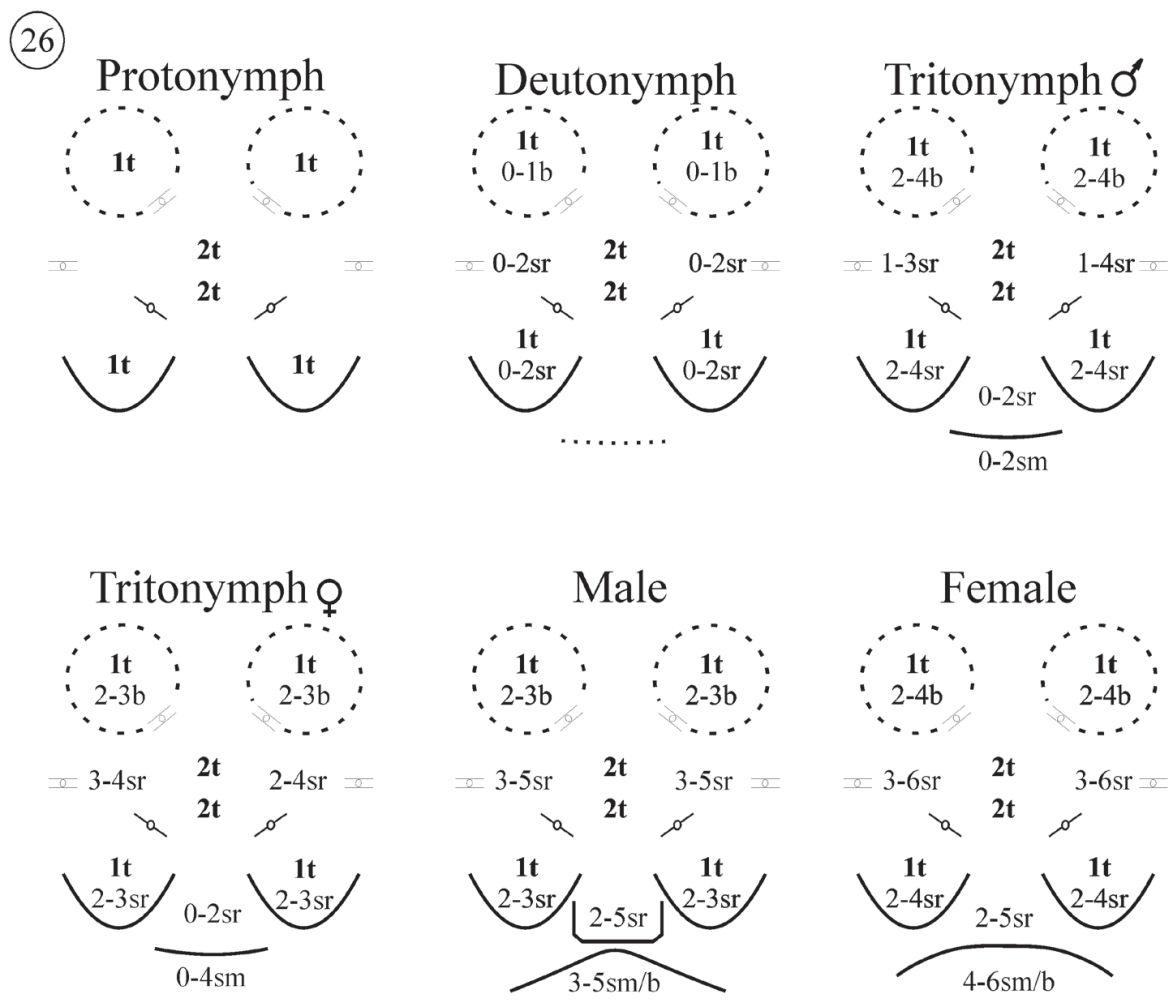
*Neocarus proteus* sp. n., schematic representation of the variation in the numbers of setae observed in the sternogenital area of nymphs and adults; sr, stout and ribbed setae; t, tapering and ribbed setae; b, tapering and barbed; sm: smooth.

*Sternal region in adults* (Figs 20–26). Sternal verrucae each with one long barbed, tapering (*St*1) and 2–4 smaller, barbed, fine setae. Remaining sternal area with two pairs of barbed, tapering (*St*2 and *St*3) and 2–5 pairs of stout, ribbed and barbed setae usually positioned more laterally.

*Pregenital and genital area of the female* (Figs 20–22 and 26). Each pregenital capsule with one barbed, tapering (*St*5) and 2–5 stout, ribbed and barbed setae. Pregenital area with 2–5 setae of different shapes: stout, ribbed and barbed or smooth. Genital setae with a fine tip, but variable in shape, smooth to barbed at the base and positioned in an invagination, hidden in most of the specimens examined. They are exposed only during partial or total evagination of the ovipositor (N = 3), when they can be observed at the base of that structure (none present at tip). Ovipositor has a tube-like shape, with two rounded structures, similar to glands, and three membranes positioned at tip. In the invaginated ovipositor these membranes remain folded, but in the evaginated ovipositor they are expanded as lobes.

*Pregenital and genital area of male* (Figs 23–26). Pregenital capsule with one barbed, tapering (*St*5) and 2–4 stout, ribbed and barbed setae. Pregenital area with 4–7 (rarely 2) stout, ribbed and barbed setae. Genital area with 2–5 small, tapering and barbed setae. Accessory glands in males include a pair of large anterior and a pair of small posterior glands. Large glands each with a small canal-like protuberance.

**Figure 27.**
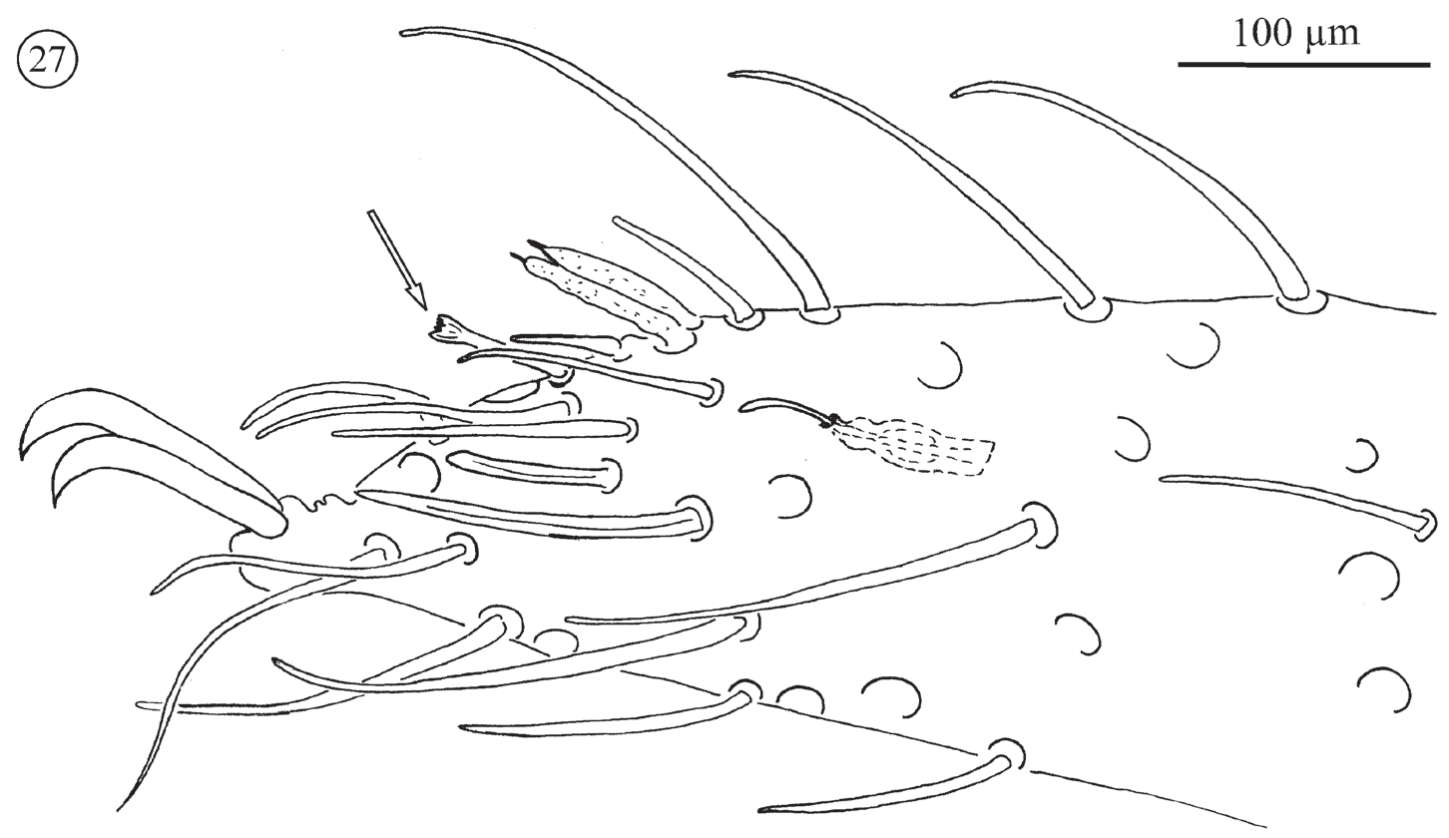
*Neocarus proteus* sp. n., anterior portion of tarsus I. Sensillum with “crown-like” tip arrowed.

**Figure 28–29.**
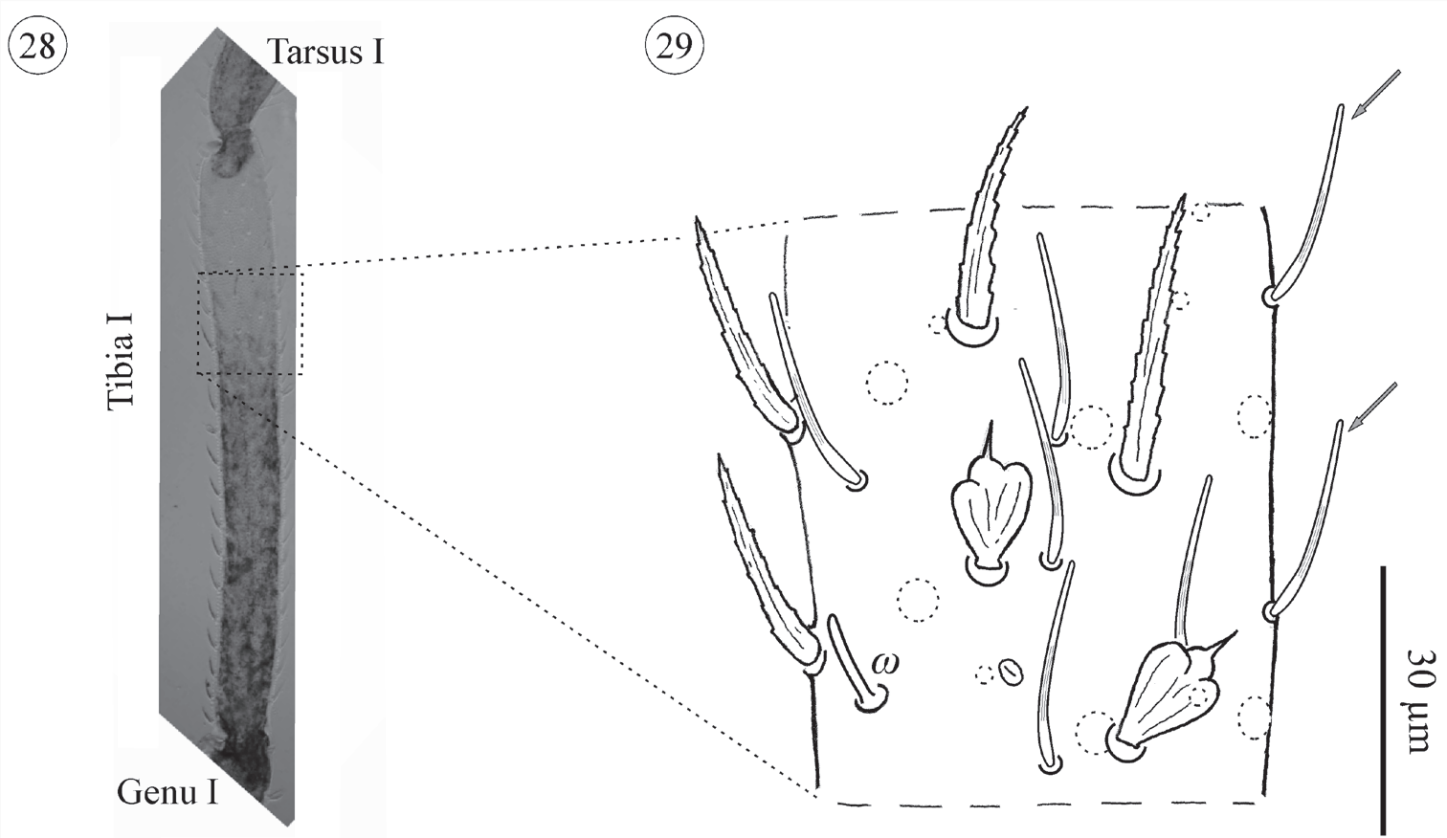
*Neocarus proteus* sp. n., tibia I of adult male. **28** General aspect of tibia I **29** details showing solenidion and smooth setae. Arrows indicate smooth setae.

**Figure 30–31.**
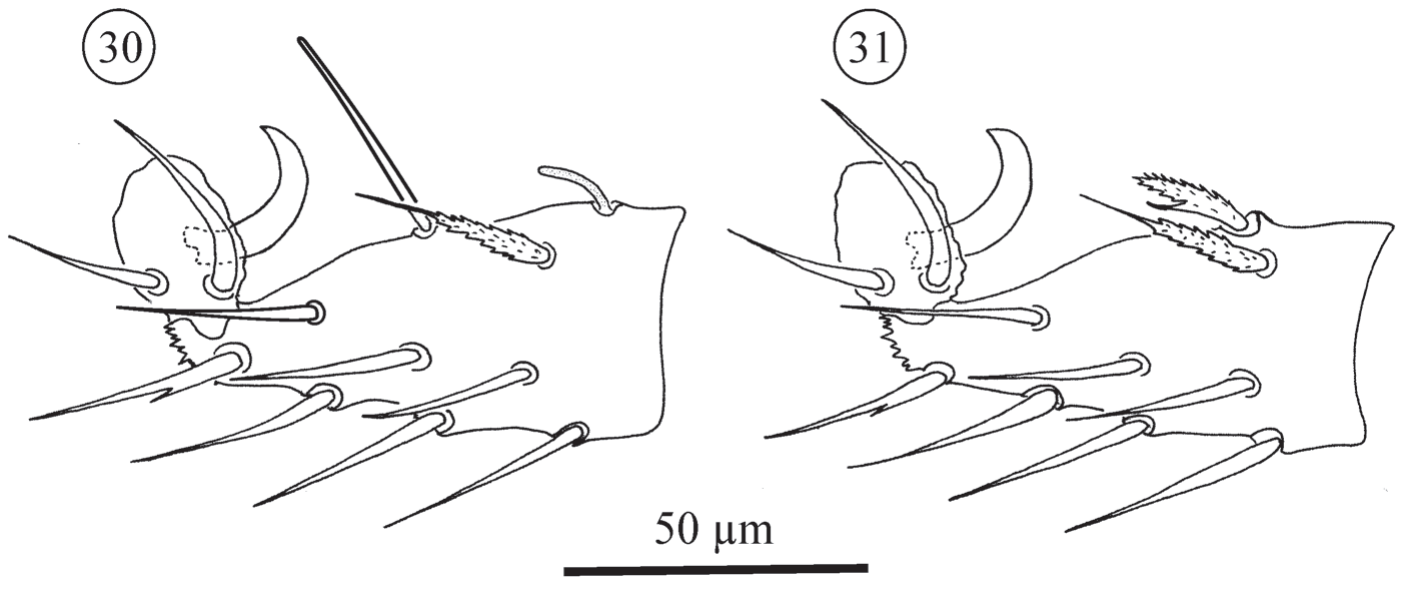
*Neocarus proteus* sp. n., adult female, acrotarsus II. **30** anterolateral view **31** posterolateral view.

**Figures 32–33.**
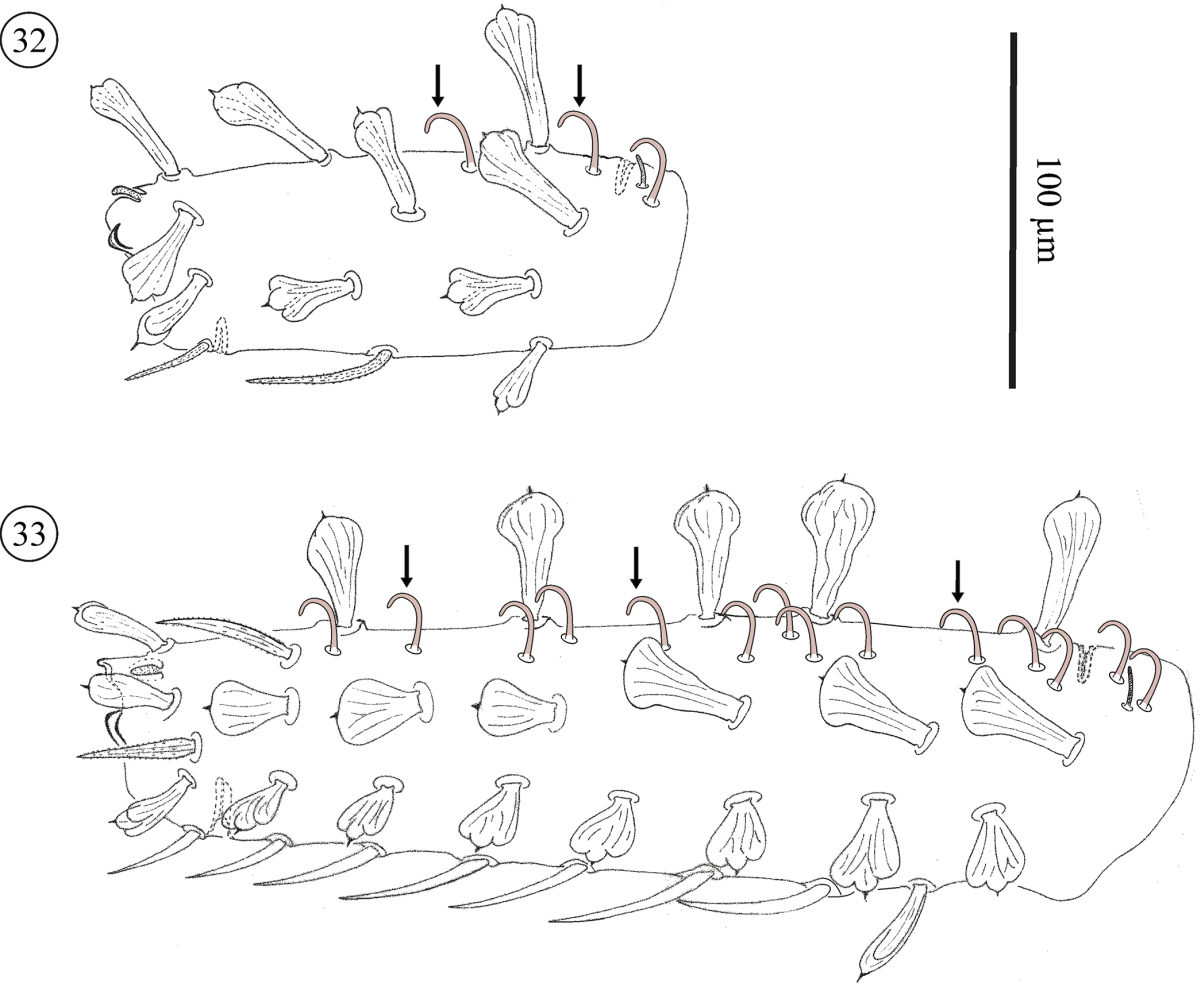
*Neocarus proteus* sp. n., anterolateral view of basitarsus IV. **32** protonymph **33** adult female. Arrows indicate some of the coronidia.

*Legs* (Figs 27–33, Tables 3 and 4). Leg I longer than others in all instars. Acrotarsi legs II–IV differentiated in all adults, all female tritonymphs, and a few male tritonymphs. Acrotarsal differentiation absent in other male tritonymphs, deutonymphs and protonymphs.

Leg I: Studies of legs I are often difficult, because these legs are fragile and often lost during collection. The results presented are based on three TN♂, three TN♀, six adult females and eight adult males. They show a type of sexual dimorphism that has not previously been recorded for Opilioacaridae. Males carry a number of smooth setae on the tibia (ranging from 29–59), genu (10–22), femur (6–21), and occasionally the trochanter (one smooth setae present in just two specimens). These setae were not observed in females. In tritonymphs this type of setae was observed in just one male tritonymph, placed on the anterior portion of the tibia.

**Table 3.** Mean length and standard deviation (µm) of the legs and palp in all instars of *Neocarus proteus* (4 PN, 9 DN, 6 TN♂, 18 males, 7 TN♀ and 13 females).

Stages	Leg I	Leg II	Leg III	Leg IV	Palp
**PN**	1080 (± 35)	654 (± 14)	634 (± 18)	1012 (± 52)	288 (± 23)
**DN**	1423 (± 37)	790 (± 42)	816 (± 42 )	1235 (± 45)	359 (± 20)
**TN**♂	1762 (± 130)	996 (± 90)	993 (± 136)	1465 (± 182)	384 (± 13)
**Adult** ♂	2282 (± 163)	1303 (± 73)	1331 (± 84)	2063 (± 182)	511 (± 27)
**TN**♀	1905 (± 243)	1069(± 88)	10889(± 82)	1704 (± 136)	431 (± 37)
**Adult**♀	2397 (± 105)	1410 (± 104)	1416 (± 94)	2246 (± 103)	533 (± 16)

Telotarsus I has a highly modified group of dorsal setae located in the apical portion, close to the tarsal claws, homologous to the Haller’s organ of ticks (Moraza 2005). Basitarsus I carries just two types of setae, only smooth setae in the distal half, and a mixture of smooth and tapering, barbed setae in the basal half. All other leg segments carry three types of setae arranged in distal to basal rows: 1) tapering and barbed, 2) papilliform and 3) smooth setae.

One solenidion is present on basitarsus I in all instars. On tibia I solenidia were not observed in the proto- and deutonymphs, appearing in the tritonymph (1–3) and adults (3–5). One caveat: the number of proto- (N = 2) and deutonymphs (N = 3) with leg I in the correct position to observe the solenidia was quite small. More specimens are needed to confirm this addition sequence.

Legs II–IV in adults: dorsal portion of acrotarsus II with a ribbed and bifurcate seta, one small solenidion, and one long and smooth sensillum (probably also a solenidion). Legs III and IV carry on the dorsal portion only 3 long and barbed setae. Additionally, acrotarsi II–IV present 3 pairs of smooth ventral setae, 1 pair of lightly barbed ventrolateral setae (positioned distally), 2 pairs of smooth lateral setae, and 1 pair of smooth laterodorsal setae (positioned distally). Pretarsi in all instars with one pair of claws and 2 pairs of setae, one pair long and curved, the other small and straight. Pretarsal ambulacrum rounded and smooth.

Coronidia (Figs 32 and 33, arrow) are present in all instars studied, but their number and distribution expands from protonymphs to adults. In protonymphs coronidia are restricted to basitarsi II–IV. In the deutonymphs coronidia appear also on tibiae II–IV. In tritonymphs and adults the distribution of coronidia extends to the genua of legs II–III. The number of the coronidia, and their position is indicated in Figures 32–33 and in Table 4. Coronidia are short and smooth, characteristics that make them similar to the setae present on legs I of the male (see above). However, coronidia in the strict sense are strongly curved middorsally, whereas the smooth setae on legs I are straight. Coronidia s.st. occur solely on legs II–IV, while the smooth setae are limited to legs I of the male.

**Table 4.** Number and position of coronidia on legs II–IV in *Neocarus proteus* (N = 4 PN, 9 DN, 6 TN♂, 18 ♂, 7 TN♀ and 13 ♀).

Stage	Leg II			Leg III			Leg IV	
	Basitarsus	Tibia	Genu	Basitarsus	Tibia	Genu	Basitarsus	Tibia
PN	2	0	0	2	0	0	2–3	0
DN	2	1–2	0	2–3	1–2	0	3–6	1–3
TN	2–4	2–6	0–2	2–4	3–6	0–2	4–9	0–7
Adult	4–8	4–8	1–4	4–9	4–8	1–4	7–15	5–10

**Figure 34.**
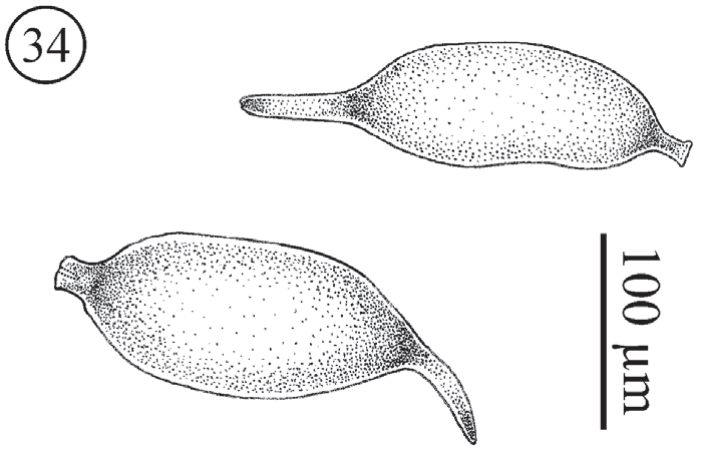
*Neocarus proteus* sp. n., eggs inside female.

*Eggs* (Figs 34); During dissection of 4 females we observed eggs inside the body. The eggs present varying sizes, suggesting different stages of maturation. The number of eggs observed inside the females was 4, 4, 5 and 6. All eggs observed in *Neocarus proteus* are similar to *Neocarus texanus* eggs, as described by Hammen (1966). They are oval, elongated and whitish in color, two different processes, one blunt and other elongated, were observed at opposite ends of the egg. The blunt process contains an invagination, but the canal described by Hammen (1966) was not observed. Notably, the processes are absent after oviposition (Klompen 2000).

##### Etymology.

Proteus comes from the adjective Greek “protean”, meaning versatile, mutable, capable of assuming many forms.

## Discussion

Sexual differentiation was not observed in the proto- and deutonymphal instars, but such differentiation was noted in tritonymphs. One of the most consistent secondary sexual dimorphisms appears to be the presence (or absence) of additional subcapitular setae with rounded tips (present in female tritonymphs and females). This traids is present in more than 12 undescribed Brazilian species and, despite not being mentioned in the original descriptions, is also present in *Neocarus potiguar* and *Caribeacarus brasiliensis*. Notably, this character is quite different from those listed by Hammen (1969) and Coineau and Hammen (1979). For example, Hammen (1969) suggested sexual differentiation in the tritonymph of *Neocarus platensis*, based on the pattern of pregenital setae (present in males, absent in females). This is unlikely to be valid for *Neocarus proteus* as setae may or may not be present in the pregenital area in tritonymphs of both sexes (they are present in that area in all adults). That character therefore has limited use for sexual differentiation of tritonymphs in *Neocarus proteus*.

**Figure 35.**
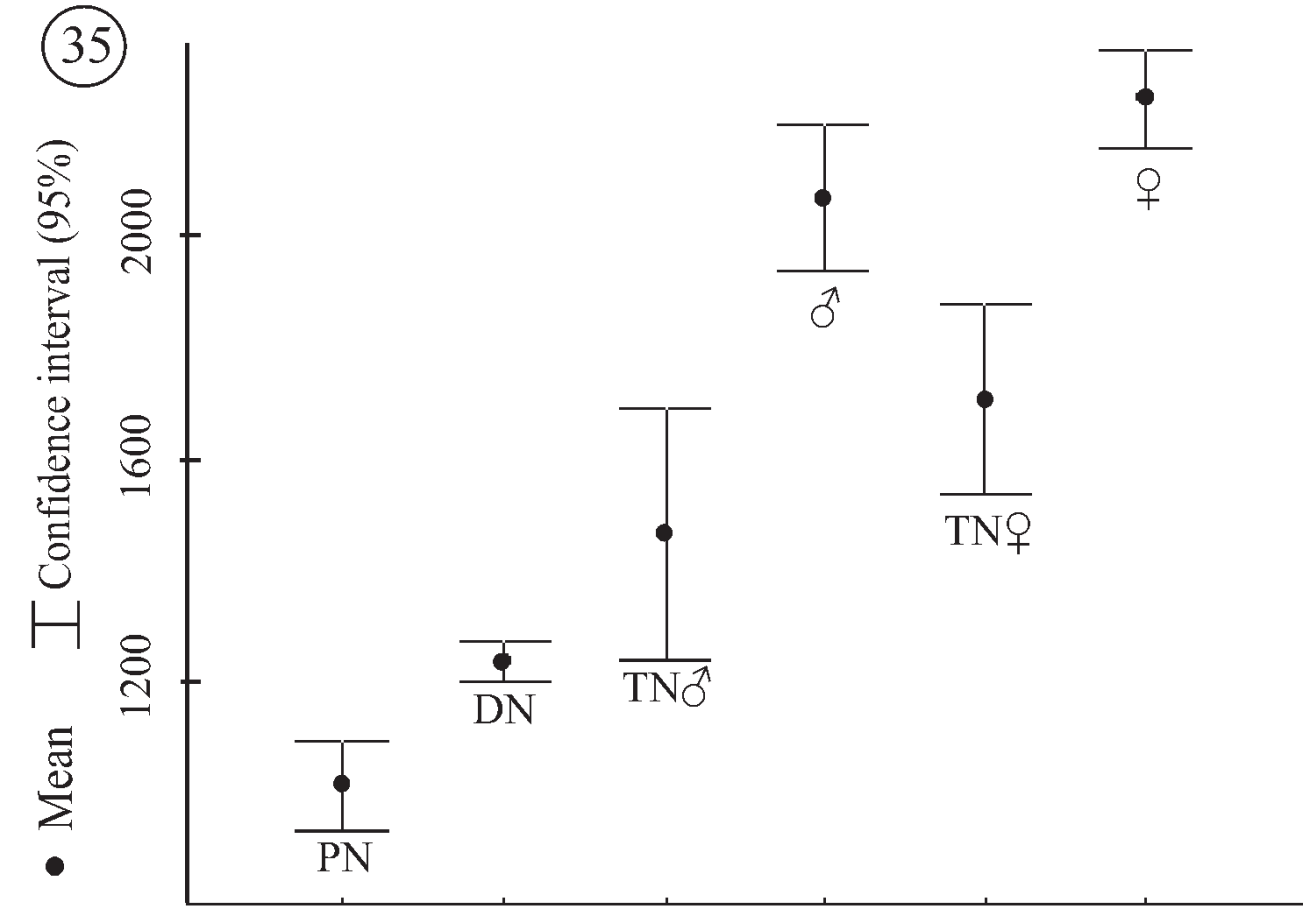
*Neocarus proteus* sp. n., length of leg IV in all instars studied.

The difference in the number of specific subcapitular setae is not the only type of sexual dimorphism expressed in tritonymphs and adults. Different patterns in the size of body parts could also be documented. Legs IV and the palps in female tritonymphs are significantly longer than in male tritonymphs (leg IV, F = 5.55, p = 0.04628; palp, F = 9, p = 0.01). This difference is due to differences in growth rate. When comparing the average size of all legs, those of female tritonymphs are 25% to 28% larger than those of deutonymphs, whereas legs of male tritonymphs are just 19% to 20% larger. These growth rate differences in tritonymphs reflect size differences for legs II–IV of males and females (data for legs I are insufficient). Legs II–IV in adult females are significantly larger than in adult males (leg II: F = 7.5, p = 0.01; leg III, F = 4.55, p = 0.046; leg IV F = 5.22, p = 0.034; palp F = 4.84, p = 0.039) (Fig. 35).

Overall size increase of the legs from protonymph to adult is about 2–2.5×. This resembles the growth pattern observed in the acariform mite families Trombiculidae and Histiostomatidae (Jones 1954), in which adults about 2.5× larger than protonymphs.

## Supplementary Material

XML Treatment for Neocarus proteus
